# Bland–Altman Analysis of Different Radiographic Measurements of the Hallux Valgus Angle and the Intermetatarsal Angle After Distal Osteotomy

**DOI:** 10.1111/os.12759

**Published:** 2020-08-27

**Authors:** Xuhan Cao, Zixing Bai, Chengyi Sun, Jianmin Wen, Xinxiao Lin, Weidong Sun

**Affiliations:** ^1^ Wangjing Hospital of China Academy of Chinese Medical Sciences Beijing China; ^2^ Beijing University of Chinese Medicine Beijing China

**Keywords:** Bland‐Altman analysis, Hallux valgus, Measurement, X‐ray films

## Abstract

**Objective:**

The aim of the present study was to evaluate commonly used approaches for detection of radiographic angles in hallux valgus deformity patients.

**Methods:**

This retrospective study was conducted in patients with hallux valgus deformity at Wangjing Hospital of China Academy of Chinese Medical Sciences from January 2016 to January 2019. The inclusion criteria were: (i) postoperative dorsoplantar weight‐bearing radiographs for the feet of patients with the hallux valgus; (ii) patients had been managed with a distal osteotomy of the first metatarsal and the osteotomized bone ends recovered. The exclusion criteria applied were as follows: (i) age > 65 years or < 18 years old; (ii) blurry image; (iii) previous history of severe foot trauma and surgery. Postoperative radiographs for hallux valgus were analyzed using six methods: by a line drawn through the long axis of the first metatarsal bone (method 1); an extended line drawn to bisect the shaft of the metatarsal at two levels with joined points of bisection (method 2); a line drawn to connect the center of the articular surface of the metatarsal head and the center of the proximal articulation (method 3); a line drawn from the center of the head of the first metatarsal head through the center of the base of the first metatarsal bone (method 4); a line drawn through the center of the head and the center of the proximal shaft (method 5); and a line drawn from the center of the head of the first metatarsal head through the center of the proximal articulation (method 6). The measurement results obtained were subjected to Bland–Altman analysis and consistency evaluation.

**Results:**

A total number of 20 radiographs were collected for measurement. No statistically significant differences were found in the measurement values among the six methods (*P* > 0.05). The lowest values of the average measurement, standard deviation, and confidence interval were established in method 3, followed by those in methods 1 and 4. The standard deviation of the measurement value and the confidence interval in method 2 were the largest. Methods 1 and 4 had similar confidence intervals and were with a high consistency. Due to the nature of the retrospective study, no follow‐up and complications were applicable in the present study.

**Conclusion:**

Line drawn through the long axis of the first metatarsal bone (method 1) and line drawn from the center of the head of the first metatarsal head through the center of the base of the first metatarsal bone (method 4) were reliable and well repeatable, and may be used for postoperative radiographs.

## Introduction

Hallux valgus angle (HVA angle) and the first intermetatarsal angle (IMA angle) are important indicators for judgment on the severity of bunion deformity and for evaluation of the bunion treatment effects. Correct measurements of HVA and IMA angles are essential for guidance and assessment of bunion therapy^1^. Approximately 200 surgical methods are applied for bunion treatment, with more than a dozen of them being commonly used surgery approaches in clinical practice, most of which correct the deformity by osteotomy of the first metatarsal bone^2^. These procedures may be used alone or in combination to correct bunion deformity. Osteotomy of the diaphysis, the base, or the fusion and reconstruction of the joint lead to deformity of the first metatarsal bone. Moreover, the resection of the osteophyte on the medial side of the first metatarsal bone was found to change the width of the distal end, hampering the determination of the axis of the first metatarsal bone^3^. The key to the proper measurement of HVA angle and IMA angle after bunion surgery lies in the determination of the axis of the first metatarsal bone. The application of various determination methods of the axis of the first metatarsal bone causes differences in the measured values of the two angles, which lowers or completely hinders the comparability of the data of different clinical studies. Graziano^4^ found that measuring the same X‐ray film after bunion treatment by different measurement methods led to considerable differences in the results obtained. Six measurement methods are usually implemented after bunion surgery, of which the center‐of‐head method is the most commonly used. Few studies exist in the literature on consistency evaluation of various measurement methods. Reliability and validity are two indicators for assessments of the accuracy or "quality" of a measurement method^5^. The results of the evaluation of the consistency of measurement methods and those of the analysis of the differences in reliability and validity between measurement methods can be used to distinguish their advantages and disadvantages and determine the best method. The Bland–Altman method is an organic combination of quantitative and qualitative analysis. In consistency evaluation, it not only considers the impact of random and systematic errors. In combination with professional significance, this approach facilitates making an objective judgment of these method properties. This method has unique advantages and can be used as a preferred method for consistency evaluation. X‐ray measurement of bunion is usually manually drawn on the X‐ray sheet. Due to that manual determination and connection of each of the reference points, its reliability and repeatability are low, which increases the measurement bias. With the advances in computer and network technologies, workstations have been installed in hospitals, which can read and measure films through the network. These devices provide convenient use of professional X‐ray film measurement tool software. Future development is expected to gradually replace manual measurement with professional tool software measurement.

The classic method of measurements of bunion and IMA angles is based on establishing the axes of metatarsal diaphysis and the proximal segment of the big toe. It is a consistent and recognized standard measurement method in preoperative measurement^6^ that can be considered the "gold standard" of preoperative X‐ray measurement. Bunion surgery leads to malunion in the osteotomy site, which hampers the accurate manual determination and measurement of the axis of the first metatarsal bone. Several methods for postoperative measurement of bunion can be clinically applied to avoid measurement errors. Professional computer measurement software enables the establishment of the axis of the first metatarsal bone in postoperative malunion. The axis determination tool provided by the measurement software facilitates the accurate drawing of the axis of the first metatarsal bone. Standard preoperative measurement method can then be applied for postoperative measurement by professional computer measurement software. This approach is the "gold standard" of software measurement that is used to evaluate the merits of other measurement methods. In this study, we defined the reliability and validity of various measurement methods through consistency evaluation of six methods of postoperative measurement of bunion.

Furthermore, we determined the most reliable measurement methods to provide reference for research on postoperative measurement and evaluation of bunion.

## Materials and Methods

### *X‐Ray Plain Film Selection*

This retrospective study was conducted in patients with hallux valgus deformity at Wangjing Hospital of China Academy of Chinese Medical Sciences between January 2016 and January 2019. The following inclusion criteria were implemented: (i) postoperative dorsoplantar weight‐bearing radiographs for the feet of patients with the hallux valgus; (ii) patients had been managed with a distal osteotomy of the first metatarsal and the osteotomized bone ends recovered. The exclusion criteria applied were as follows: (i) age > 65 years or <18 years old; (ii) blurry image; (iii) previous history of severe foot trauma and surgery. The images were output from the CR (Computed Radiography) image workstation until stored into JPG (Joint Photographic Experts Group）pattern.

### *Definition of the Radiographic Angles*

HVA was termed as the angle between the first metatarsal bone and the proximal phalanx of the first toe axis.

IMA was defined as the angle between the first and second metatarsal axis.

### *Measurement of the Radiographic Angles—First Metatarsal Axis*

**Fig. 1:**
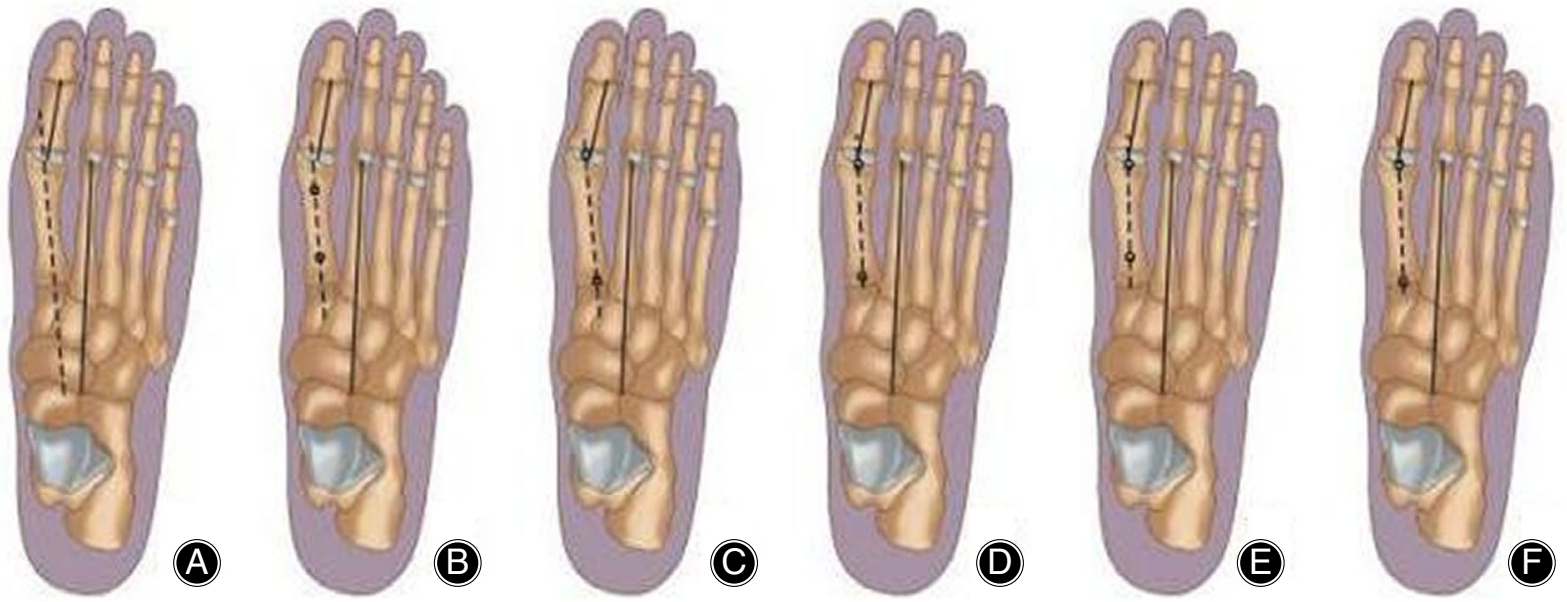
This figure shows six methods to determine the first metatarsal axis of X‐ray film after distal pollicis valgus osteotomy. Six different measurement methods will lead to the deviation of the evaluation of the postoperative effect. Method A: The first metatarsal axis passes through the first metatarsal long axis. Method B: Connect two bisection points on different levels of metatarsal stem. Method C: Connect the middle points of the distal and proximal articular surfaces of the first metatarsal bone. Method D: The line between the central point of the first metatarsal head and the central point of the base of metatarsal. Method E: The line between the first metatarsal head and two central points of the proximal metatarsal shaft. Method F: The line between the central point of the first metatarsal head and the central point of the joint surface near the first metatarsal.

The radiographs were analyzed using the Image Pro Plus 6.0, and the data were saved in Excel^7^, ^8^, ^9^. Before measuring the radiographic angles, the experienced medical staff marked the axis of the second metatarsal bone and the proximal phalanx of the first toe. Then, the longitudinal axis of the first metatarsal bone was independently measured. In the same measurement, we drew the axis of the first metatarsal bone in the radiographs using the six different methods presented below. The measurements in each method were repeated at least three times. After at least 1 week, the above process was repeated. Measurements were performed by five independent teams of the staff in three radiographs for at least three times. Similarly, in an at least 1 week interval, the measurement was repeated one more time. The measurement procedures are listed below **(**Fig. 1 **)**. Method 1: A line was drawn through the long axis of the first metatarsal bone **(**Fig. 1A **)**. Method 2: A line was drawn to bisect the shaft of the metatarsal bone at two levels, with joined points of the bisection and an extended line **(**Fig. 1B **)**. Method 3: A line was drawn to connect the center of the articular surface of the metatarsal head and the center of the proximal articulation **(**Fig. 1C **)**. Method 4: A line was drawn from the center of the first metatarsal head through the center of the base of the first metatarsal bone **(**Fig. 1D **)**.

**Fig. 2:**
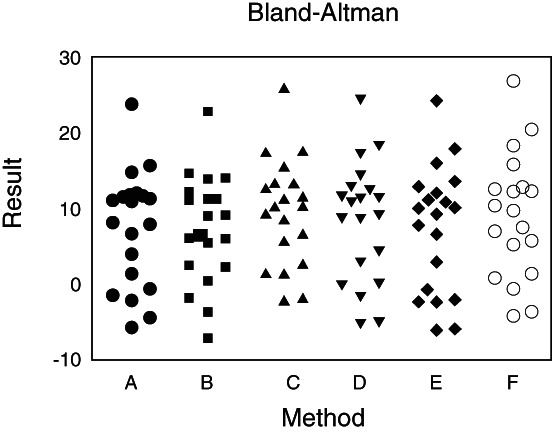
This figure shows a surveyors using these six methods to measure the X‐ray film after the distal hallux valgus osteotomy with the help of Image Pro Plus 6.0 software. The measured results were analyzed with SAS 9.1.3 statistical software data package. The Bland–Altman method was used to calculate the difference and mean value of six measurement methods, and the Bland–Altman diagram was drawn with medcalc 12.3.0.0 software to observe the relationship between the difference and mean value.

**Fig. 3:**
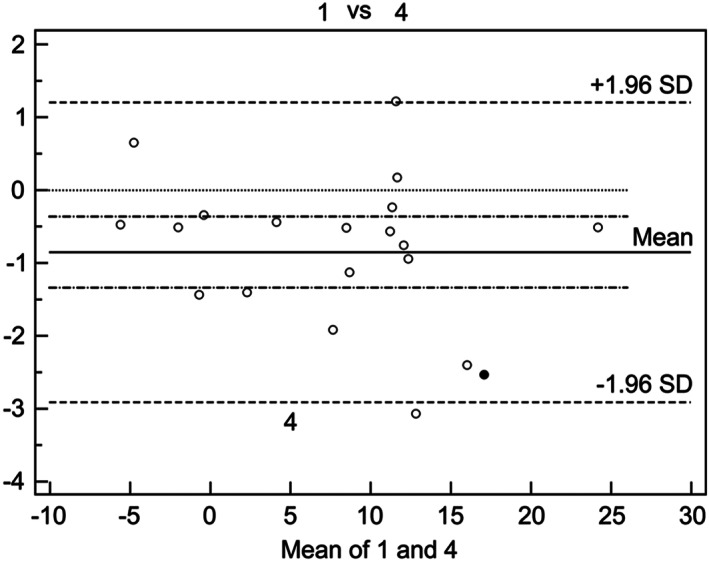
According to the analysis data, method 1 is consistent with method 4. In this paper, the Bland–Altman method is used to draw the scatter diagram with the difference between method 1 and method 4 as the ordinate and the average value as the abscissa. The scatter diagram of method 1 and other methods are drawn in the same way. It can be seen that method 1 and method 4 have the lowest confidence interval and the highest consistency.

Method 5: A line was drawn through the center of the head and the center of the proximal shaft **(**Fig. 1E **)**. Method 6: A line was drawn from the center of the first metatarsal head through the center of the proximal articulation **(**Fig. 1F) Fig. 2, 3.

### *Standardizing Measurement Technique of the Radiographic Angles—the Other Axes*

The reference points to identify the axis of the second metatarsal bone were localized in the metaphyseal diaphyseal region 1–2 cm proximal to the distal articular surfaces and 1–2 cm distal to the proximal articular surface^9^.

The reference points for identification of the axis of the proximal phalanx first toe were localized in the metaphyseal diaphyseal region 0.5–1.0 cm proximal to the distal articular surface and 0.5–1.0 cm distal to the proximal articular surface.

A geometric device (the Mose sphere) was used to determine the center of the best‐fit circle corresponding to the first metatarsal head. The latter was defined as the circle with three points of contact, namely, the lateral edge, the outer edge, and the top of the first metatarsal head^8^.

Measurement variability served as quality control. The following criteria were used: interobserver variability of the measurements of HVA and IMA with a deviation of late intra‐observer variability of the measurements of HVA and IMA was set with a deviation of ≤4°.

### *Statistical Analysis*

SAS 9.1.3 (Statistical Analysis System, NCSU, USA) was used for data analysis. The Bland–Altman method was applied for calculating the difference and mean. Medcalc 12.3.0.0 (MedCalcSoftware, Ostend, Belgium) was employed for drawing the Bland–Altman diagrams to evaluate the relation between the difference and the mean. If the difference of the two methods was within the consistency limits, it was clinically acceptable and denoted as good consistency. *P‐*values less than 0.05 were considered statistically significant.

## Results

### *No Significant Differences Were Detected Among the Interobservers*

Five measurements were performed on a total number of each of the 20 radiographs using six methods. The differences among the measurements were comparable (*P* > 0.05). The first metatarsal axis is key to the measurements of HVA and IMA. Hence, the differences in the measurements of HVA and IMA are consistent. Data of HVA are presented in Table 1.

**Table 1:** Differences in the measurements of HVA among different measurers (mean ± SD)

Measurement method	Measurer	HVA (°)	F	*P*
A		10.49 ± 3.93		
	2	11.06 ± 4.07		
	3	9.52 ± 3.73	0.51	0.487 3
	4	9.70 ± 3.58		
	5	8.99 ± 3.57		
B	1	8.82 ± 2.70		
	2	10.69 ± 3.17		
	3	8.91 ± 2.68	0.38	0.546 9
	4	8.72 ± 3.28		
	5	11.29 ± 1.53		
C	1	10.77 ± 3.30		
	2	11.32 ± 3.71		
	3	12.56 ± 4.63	0.32	0.583 1
	4	12.44 ± 4.77		
	5	12.09 ± 4.11		
D	1	9.87 ± 3.98		
	2	10.50 ± 4.11		
	3	10.26 ± 4.41	0.01	0.920 8
	4	10.66 ± 4.24		
	5	9.47 ± 2.52		
E	1	9.52 ± 4.59		
	2	10.41 ± 4.64		
	3	9.55 ± 4.61	0.01	0.935 0
	4	9.91 ± 4.60		
	5	9.48 ± 2.79		
F	1	10.45 ± 3.80		
	2	11.13 ± 3.46		
	3	10.77 ± 3.66	0.01	0.924 1
	4	10.84 ± 3.85		
	5	10.33 ± 2.41		

HVA, it was termed as the angle between the first metatarsal bone and the proximal phalanx of the first toe axis. *P* value, which is an index to measure the difference between the control group and the experimental group, means *P* < 0.05, indicating that there is significant difference between the two groups, *P* < 0.01, indicating that the difference between the two groups is extremely significant. F value, which is used to evaluate the difference between groups. It is also used to represent the significance of the whole fitting equation. The larger f is, the more significant the equation is and the better the fitting degree is.

### *Analysis of Consistency of the Five Measures Using the Six Different Methods*

Our analysis of the agreement of the measurements of the intra‐observers revealed the lowest mean values and confidence interval in method 3, followed in an ascending order by methods 1 and 4, which ranked the second. The mean values and confidence interval of method 2 were the highest.

In the assessment of the agreement of the intra‐observer measurements of the six methods, we found that method 1 and method 4 had the lowest mean values and confidence intervals. Similar results were obtained in the measurements of IMA using these six methods.

## Discussion

In the present study, we established that line drawn through the long axis of the first metatarsal (method 1) and the line drawn from the center of the head of the first metatarsal head through the center of the base of the first metatarsal (method 4) were reliable and well repeatable. Therefore, they can be used for assessment of postoperative clinical radiographs.

### *Current Research Status of the Management of the Relative Radiographic Angles in Postoperative with Hallux Valgus*

Many surgical treatment strategies exist for the hallux valgus correction, most of which correct deformity by osteotomy of the first metatarsal. The use of different methods may lead to substantial differences in the radiographic measurements of HVA and IMA due to variability in the reference points defining the longitudinal axis of the first metatarsal bone^9^. Thus, there is no comparability of different clinical research data. The measurement methods for identification of the axis, described in the available literature, can be classified into three groups^10^:measurements of mid‐diaphyseal points distally and proximally, measurements of the center of the head and the center of the base points, and such of the center of the distal and proximal articular surfaces^3^. Schneider *et al*.^10^ performed measurements of weight‐bearing radiographs using five methods (methods 1 to 5) in 20 preoperative and postoperative patients managed by distal Chevron osteotomy. They found small differences in the measurements of the radiographic angles using the five methods before operation, but considerable differences when they were applied postoperatively. These authors evaluated the measurement accuracy to define the longitudinal axis of the first metatarsal bone. Other scholars^11^ considered that the most accurate measurement of HVA and IMA in postoperative patients was the one by a distal osteotomy of the first metatarsal bone using method 4. Srivastava *et al*.^10^ also recommended method 4 for radiographic measurements of the hallux angles. Importantly, method 4 could be used for the measurement of HVA and IMA in patients managed by Chevron and Scarf osteotomy. Allen *et al*.^12^ retrospectively analyzed the measurement of IMA in patients with proximal osteotomy of the first metatarsal bone. The researchers found that method 4 was used in very few of the radiograph measurements. Moreover, they found significant differences between that method 4 and method 1 which was used in most radiographs. Thus, they concluded that method 4 was more accurate than method 1. Shima *et al*.^8^ measured radiographs through five different methods (method 2 to method 6) in patients with crescentic metatarsal osteotomy They found that method 2 and method 5 had poor reliability. The difficulty of defining the articular margins might have led to low reliability of method 3. In method 4, the proximal reference point is placed near the osteotomy site of the first metatarsal, conversely, in method 6, the center of the proximal articular surface is not influenced by the osteotomy site. Therefore, they concluded that method 6 was more reliable than method 4. Additionally, Kopp *et al*.^13^ measured radiographs in the HVA and IMA of patients as previously outlined by Coughlin *et al*.^14^ using the modified Lapidus procedure.

### *Establishment of a Gold Standard of the Postoperative Radiograph Measurements*

The classical measurement (method 1) of HVA and IMA was based on the long axis of the first metatarsal and was the accepted preoperative gold standard. However, deformity of the first metatarsal bone caused postoperatively by osteotomy hampered performing manual measurements for determination of the axis of the first metatarsal bone, leading to erroneous results. Various measurements have been used in clinical radiography in attempts to avoid errors^7^, ^8^, ^9^. It was essential to easily, repeatedly, and accurately determine the reference point and avoid the influence of osteotomy and anatomy variation of the first metatarsal bone. Professional measurement software allowed easy determination of the axis of the first metatarsal bone of the postoperative radiographs and exclusion of the axis of the first metatarsal bone that was influenced by the deformity. Moreover, this software created opportunities for higher reliability and repeatability. For method 1 being confirmed as preoperative gold standard and the software determined the axis of the first metatarsal reliably, method 1 can be as the postoperative gold standard to assess another measurements.

### *Bland–Altman* *Analysis and Agreement Evaluation of Different Radiograph*

The Bland–Altman statistical analysis initially calculated the 95% limit of agreement of the two different methods. Then, if the limit could be accepted in clinical settings, we could consider that the two methods are in agreement and interchangeable.

#### *Inter‐observer Agreement Analysis*

The differences in the values obtained by each observer that used the six different methods were not statistically significant (*P* > 0.05). The six methods were reliable for the measurement of the radiographs, and no significant difference was present in terms of inter‐observers; the quality control was also valid. Therefore, we conclude that all six methods were easy to apply, and their errors were insignificant.

#### *Intra observer Agreement Analysis*

Method 2 determined the reference point in the metaphyseal–diaphyseal region that was 1–2 cm distal to the proximal articular surface. Because the reference point was influenced by osteotomy, it was difficult to obtain uniform IMA and HVA. Compared with method 1, here, the confidence interval was larger than other methods, and the consistency was the worst, indicating method 2 was the least reliable. In method 3, the longitudinal axis corresponds to the line connecting the center of the articular surface of the metatarsal head and the center of the proximal articulation. The ranges of the standard errors and confidence intervals of the two measurement values were narrow, and the reliability was high. However, the confidence interval was larger than that in method 1, compared with method 4. The reasons for the poor consistency were that the lateral edge and the outer edge were difficult to determine as Schneider^7^, ^8^ proposed. The major reason was that distal osteotomy "lied down," and the articular surface deviated from the center line, which influenced the accuracy of the measurement of the axis. Method 4, in which the longitudinal axis corresponded to the line connecting the centers of the first metatarsal head and the base, was determined by the Mose sphere. It was not influenced by the osteotomy site and was also repeatable and easy for manual determination. Method 4 has been the preferred method for manual measurement, which is consistent with the conclusion of many earlier studies^7^, ^12^. In method 5, the longitudinal axis corresponded to the line connecting the center of the head and the center of the proximal shaft. In this method, one reference point was the same as that in method 4. Thus, their agreement was higher than that with other methods, but the reference point of the proximal shaft was not only, it influenced the measurement's accuracy and repeatability. Method 6, the longitudinal axis corresponded to the line connecting the center of the head of the first metatarsal head and the center of the proximal articulation; it was a combination of method 3 and method 4. The confidence interval was larger than that in methods 1 and method 4, and the agreement worse than that with method 4. Method 6 appropriated to measure the proximal osteotomy of the first metatarsal bone as Shima *et al*.^8^ reported and this may be the reason of the poor reliability of distal osteotomy.

### *The Prospect of the Measurement of Postoperative Radiographs of the Patient with Hallux Valgus*

Manually measuring the angles was accompanied with many errors and obstacles, such as difficulty in drawing lines and corrections, and errors in measuring angles using a protractor. The professional measurement software auto‐generated the axis, easily determined the marking points, and minimized the influence of other factors. Moreover, it was reliable and repeatable. Therefore other measurement methods may not be used, except the golden standard^15^.

Some limitations of this study need to be acknowledged. First of all, only 20 radiographs were evaluated. In addition, we failed to trace the surgical or intervention procedures. Hence, subgroup or other analyses could not be performed. Moreover, dozens of means were available of the post‐operative measurements, but we included only six methods, which is not sufficient to draw reliable conclusions.

Further prospective studies are necessary to determine applicable and feasible post‐operative strategies for determination of HVA and IMA.
